# Development of the Human Placenta and Fetal Heart: Synergic or Independent?

**DOI:** 10.3389/fphys.2018.00373

**Published:** 2018-04-12

**Authors:** Graham J. Burton, Eric Jauniaux

**Affiliations:** ^1^Department of Physiology, Development and Neuroscience, Centre for Trophoblast Research, University of Cambridge, Cambridge, United Kingdom; ^2^Faculty of Population Health Sciences, EGA Institute for Women's Health, University College London, London, United Kingdom

**Keywords:** placenta, fetal heart, umbilical circulation, pregnancy, congenital heart disease, vascular resistance

## Abstract

The placenta is the largest fetal organ, and toward the end of pregnancy the umbilical circulation receives at least 40% of the biventricular cardiac output. It is not surprising, therefore, that there are likely to be close haemodynamic links between the development of the placenta and the fetal heart. Development of the placenta is precocious, and in advance of that of the fetus. The placenta undergoes considerable remodeling at the end of the first trimester of pregnancy, and its vasculature is capable of adapting to environmental conditions and to variations in the blood supply received from the mother. There are two components to the placental membranes to consider, the secondary yolk sac and the chorioallantoic placenta. The yolk sac is the first of the extraembryonic membranes to be vascularized, and condensations in the mesenchyme at ~17 days post-conception (p.c.) give rise to endothelial and erythroid precursors. A network of blood vessels is established ~24 days p.c., with the vitelline vein draining through the region of the developing liver into the sinus venosus. Gestational sacs of early pregnancy failures often display aberrant development of the yolk sac, which is likely to be secondary to abnormal fetal development. Vasculogenesis occurs in the villous mesenchyme of the chorioallantoic placenta at a similarly early stage. Nucleated erythrocytes occupy the lumens of the placental capillaries and end-diastolic flow is absent in the umbilical arterial circulation throughout most of the first trimester, indicating a high resistance to blood flow. Resistance begins to fall in the umbilico-placental circulation around 12–14 weeks. During normal early pregnancy the placental capillary network is plastic, and considerable remodeling occurs in response to the local oxygen concentration, and in particular to oxidative stress. In pregnancies complicated by preeclampsia and/or fetal growth restriction, utero-placental malperfusion induces smooth muscle cells surrounding the placental arteries to dedifferentiate and adopt a proliferative phenotype. This change is associated with increased umbilical resistance measured by Doppler ultrasound, and is likely to exert a major effect on the developing heart through the afterload. Thus, both the umbilical and maternal placental circulations may impact on development of the heart.

## Introduction

The placenta and the fetal heart are two of the first organs to differentiate, and hence it is assumed that their development is interlinked. Common genes and micronutrients, such as folate, regulate essential steps in the formation of both organs, and so cardiac and placental abnormalities frequently co-exist (Linask, 2013). However, there is mounting evidence that primary defects in placental development may influence the development of the fetal heart and its function after delivery.

Conceptually, the influence of the placenta may be two-fold. Firstly, the effectiveness of the placenta as a source of oxygen and nutrients, and as a selective barrier to xenobiotics, can have a profound impact on the morphogenesis and functional capacity of many organ systems through developmental programming (Burton et al., 2016). Secondly, despite a lack of experimental data it is highly probable that the haemodynamics of the umbilico-placental circulation impact on fetal cardiac development (Linask et al., 2014). The placenta is the largest of the fetal organs, and at term receives ~40% of fetal cardiac output. The resistance offered by the arterial and capillary network within the placental villous trees will vary according to the stage of development and the presence of placental pathology. Since cardiac gene expression is highly sensitive to biomechanical cues, that resistance may influence the differentiation of cardiomyocytes and morphogenesis of the heart (Hove et al., 2003; Kowalski et al., 2014).

In this review, we focus on the potential biomechanical cues offered by the extra-embryonic circulations that might synergise human placental and cardiac development. We concentrate on the anatomical and physiological development of the vitelline and chorioallantoic placental circulations, and consider how the haemodynamics of the fetal placental circulation, as assessed *in vivo* with Doppler ultrasound, provide further information on the potential impact of placental pathologies on umbilical haemodynamics.

## Development of the fetal-placental circulations

The extracorporeal circulation to the extra-embryonic membranes comprises two circulations, that to the secondary yolk sac, the vitelline circulation, and that to the definitive placenta, the chorionic or umbilical circulation. Of these, the vitelline circulation is the first to develop, and its maximal function is contemporaneous with morphogenesis of the heart (Jones and Jauniaux, 1995; Gittenberger-de Groot et al., 2013).

**Figure 1:**
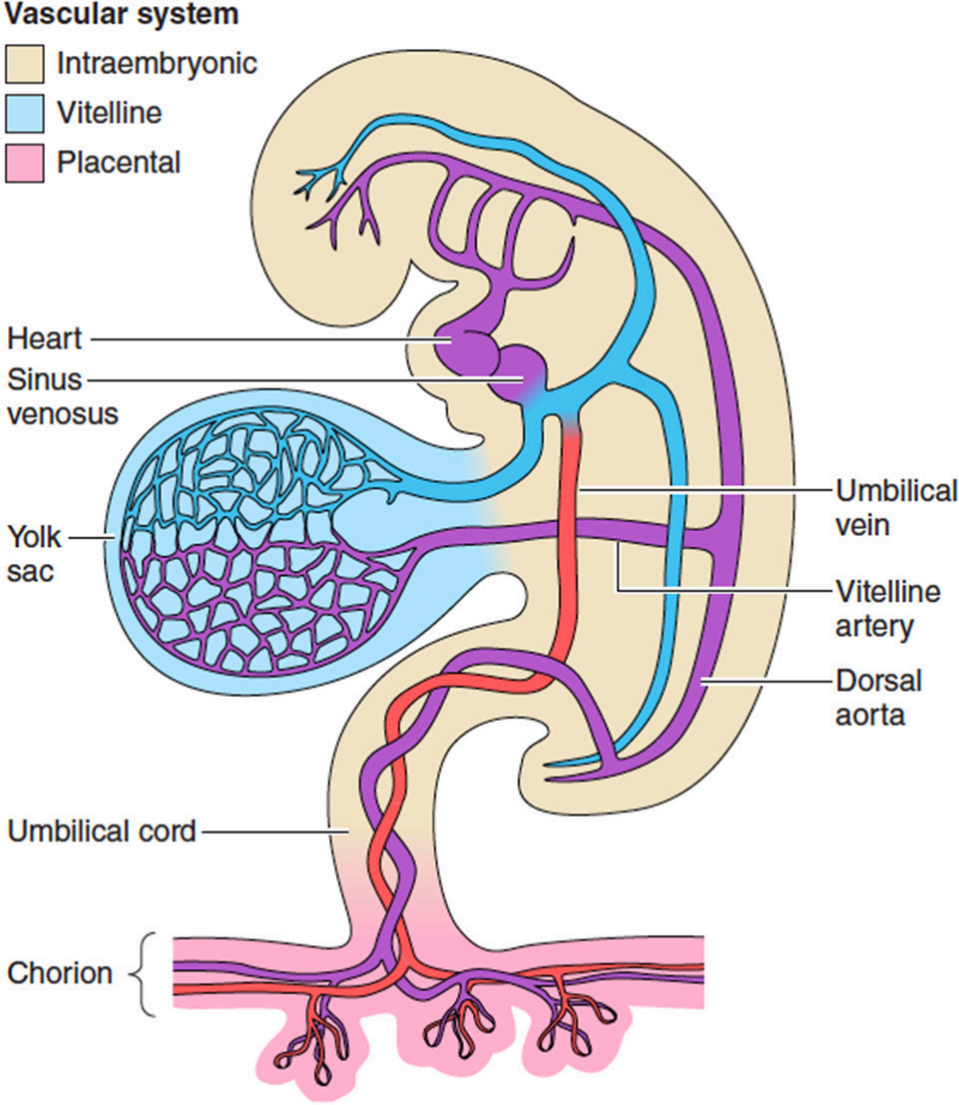
The extravillous circulations. The yolk sac is the first of the extraembryonic membranes to be vascularized, and likely plays a key role in maternal-fetal transport during the period of organogenesis before the chorionic circulation is fully established at ~12 weeks. Changes in the resistance offered by each circulation may affect gene expression and differentiation of the fetal cardiomyocytes. From Burton et al. (2016) with permission.

A capillary network can be identified within the mesenchymal layer of the human yolk sac from ~5 weeks gestational age (Pereda and Niimi, 2008), and venous drainage is through the region of the developing liver into the sinus venosus (Figure 1). The size of these capillaries remains below the resolution of standard ultrasound imaging during the biological life of the secondary yolk sac, and only the larger vessels on the vitelline duct have been studied *in utero* with color Doppler imaging toward the end of the first-trimester when it is no longer functional (Mäkikallio et al., 2004). The yolk sac shows degenerative changes from 10 weeks of gestation suggesting that its involution in normal pregnancies is a spontaneous event rather than the result of mechanical compression by the expanding amniotic cavity (Jauniaux et al., 1991d). In early fetal demise, the yolk sac increases in size and becomes less dense due to oedema just before or immediately after activity of the fetal heart has stopped. These variations in size and appearance of the yolk sac are the consequence of abnormal fetal development or death rather than being the primary cause of the early pregnancy failure (Jauniaux et al., 1991d).

The biological functions of the human yolk sac have rarely been studied (Gulbis et al., 1998), and thus are poorly understood. Recent RNA-Seq data indicate through conservation of transcripts across species that it may be important for transport of nutrients to the early fetus during early gestation (Cindrova-Davies et al., 2017). In particular, transcripts encoding proteins involved in the handling and metabolism of cholesterol are some of the most abundant. Cholesterol is essential for the formation of cell and organelle membranes, and hence cell replication, but it is also an essential co-factor for signaling molecules, such as sonic hedgehog, that play critical roles during morphogenesis (Lewis et al., 2001). As well as transport of macro- and micronutrients, the yolk sac also expresses many ATP-binding cassette (ABC) transporters that may play an important role in protecting the developing embryo during the critical period of organogenesis through the efflux of environmental toxins and xenobiotics.

Elements of the chorionic circulation can first be observed in the mesenchyme of placental villi during the 5th week of gestation. Haemangioblastic clusters differentiate and give rise to an extensive network of capillaries lying predominantly just under the trophoblastic basement membrane (Demir et al., 1989; Charnock-Jones and Burton, 2000; Aplin et al., 2015). The number of capillary profiles per villus profile, and the percentage of the villous stromal core occupied by the capillaries, increase steadily from weeks 6 to 15 of pregnancy (Jauniaux et al., 1991a). The early capillaries possess a relatively low coverage of pericytes, suggesting that they are plastic and capable of remodeling (Zhang et al., 2002).

Extensive remodeling occurs toward the end of the first trimester when the definitive placenta is formed. Villi initially develop over the entire gestational sac, but starting from around 8 weeks of gestation the villi over the superficial pole begin to regress, forming the smooth membranes or chorion laeve. Regression is associated with the progressive onset of the maternal arterial circulation to the placenta, first in the periphery and then in the rest of the placenta. This process is mediated by the migration of extravillous trophoblastic cells (EVT) into the placental bed and modulated by locally high levels of oxidative stress within the villi (Jauniaux et al., 2003a). Consistent with this theory, the junctional complexes between endothelial cells forming the capillaries within the regressing villi loose their integrity, and the villi become avascular, hypocellular ghosts (Burton et al., 2010).

Events at this stage of development play a key role in determining the final size and shape of the placenta (Burton et al., 2010; Salafia et al., 2012), and so may impact development of the fetal heart. Excessive or asymmetric regression of the villi may lead to more ellipsoid placentas or eccentric insertion of the umbilical cord, the latter being less efficient as estimated by the fetal/placental weight ratio (Yampolsky et al., 2009). Reduced efficiency may restrict the supply of nutrients to the fetus, but in addition the branching pattern of the chorionic arteries as they radiate out over the chorionic plate will be different depending on the position of the insertion of the cord. Predicting the impact of these differences on the resistance offered by the network is complex. When cord insertion is central the branching pattern of the arteries is predominantly dichotomous, whereas when the cord insertion is eccentric the monopodial pattern of branching dominates (Gordon et al., 2007). Modeling reveals that energy losses are least at monopodial branch points, and hence this pattern may be favored to ensure even distribution of blood flow when there are relatively long distances across the chorionic plate to be traversed (Gordon et al., 2007). In most placentas, however, the branching pattern is a mix of the two types, making prediction of the vascular impedance offered by the chorionic arteries difficult.

Elaboration of the peripheral components of the villous trees, the intermediate and terminal villi, increases exponentially from around 20 weeks of gestation onwards (Jackson et al., 1992). The fetal capillary network develops commensurately, and at term comprises ~550 km of capillaries, and contains ~35 mls of fetal blood (Burton and Jauniaux, 1995). The villi are arranged into 30–40 fetal lobules, each of which is supplied with maternal blood by a spiral artery and represents an independent maternal-fetal exchange unit.

Within the terminal villi, there are numerous connections between individual capillaries (Jirkovská et al., 2008; Plitman Mayo et al., 2016a). These connections create a number of seemingly parallel circuits, and it is possible that flow moves in different directions at different times according to local pressure differentials. Modeling suggests that the direction of flow through the network has little impact on the efficiency of diffusional exchange (Plitman Mayo et al., 2016b). Localized dilatations of the fetal capillaries, referred to as sinusoids, occur along their length, particularly at the points of sharp bends. It has been suggested that the sinusoids serve to reduce the resistance within a capillary loop, and thereby ensure even perfusion within a villus or series of villi (Kaufmann et al., 1988). They may have other functions, however, for the sinusoids bring the outer wall of their capillary into close contact with the inner surface of the trophoblastic epithelium covering of the villi, which is locally thinned. As a result, the villous membrane separating the maternal and fetal circulations is extremely attenuated, and these sites, referred to as vasculo-syncytial membranes, are the most important locations for diffusional exchange (Plitman Mayo et al., 2016b). The local increase in capillary cross-sectional area also leads to a slowing in flow velocity, facilitating exchange.

## Onset of the chorionic circulation

The embryonic heart starts as a primitive tube and the first contractions are seen at ~22 days (beginning of the 5th week after the last menstrual period). The heart starts to beat before the development of the conduction system, and before a competent valvular mechanism has formed (Collins, 2016). The primitive bilateral aortae, each consisting of ventral and dorsal parts, fuse during the 4th embryonic week (6 weeks LMP) to form a single definitive descending aorta. The umbilical arteries connect to the primitive dorsal aorta (Figure 1). Cardiac output and heart rate increase in proportion with the developing embryonic body. By 10 weeks the fetal heart rate reaches its peak at around 170 beats/min (bpm) and then slows down to 120–160 bpm for the rest of pregnancy (van Heeswijk et al., 1990). Abnormally slow (Doubilet and Benson, 2005) and fast (Doubilet et al., 2000) heart rates during the second month of pregnancy have been associated with a high risk of embryonic demise. It is hypothesized that subsequent hypoperfusion of the secondary yolk sac, causing a progressive loss of structure and necrosis or oedema can explain why in a pregnancy destined to miscarry, changes in the diameter of the sac may precede arrest of the embryonic heart by a few days (Datta and Raut, 2017).

**Figure 2:**
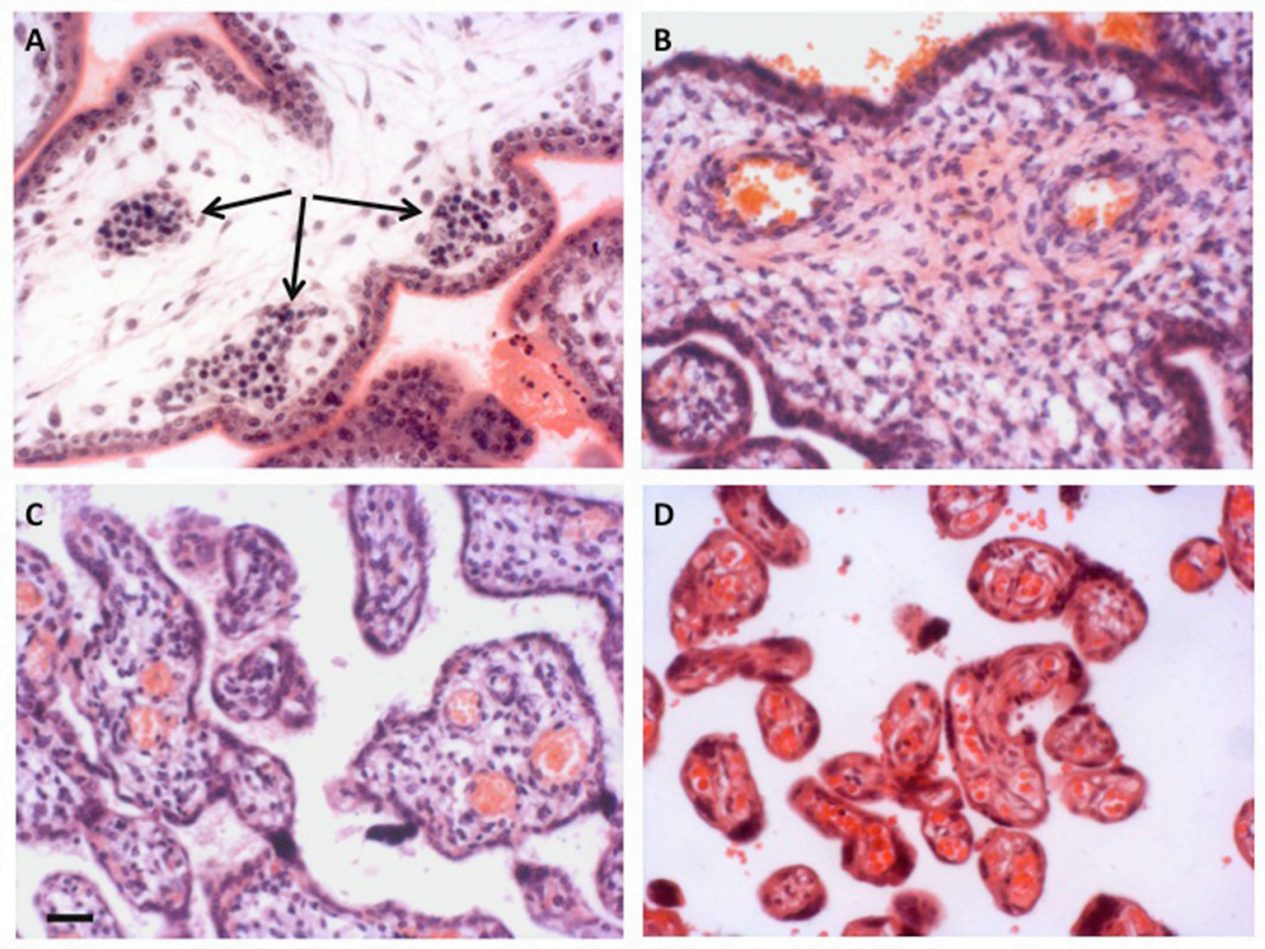
Development of the placental vasculature. **(A)** Placental villi of 6 weeks gestational age prior to onset of the chorionic circulation, showing the presence of nucleated erythrocytes in the developing fetal capillaries (arrowed). **(B)** Villi at 14 weeks gestational age showing the presence of non-nucleated erythrocytes in the larger vessels within the stromal core, indicative of onset of the chorionic circulation. **(C)** Villi of 27 weeks gestational age. By now the smaller peripheral villi are being elaborated. **(D)** Villi of 40 weeks gestational age showing well vascularized terminal villi. Scale bar for all images = 50 μm. Stain; haematoxylin and eosin for all.

Although an extensive capillary network is visible within the early villi, there is little evidence of an effective chorionic circulation during the first trimester. The capillaries remain of small caliber, and are filled with large nucleated fetal erythrocytes emerging from the secondary yolk sac that are densely packed together (Figure 2). The presence of the nuclei renders the erythrocytes less deformable than their mature forms, and thus the blood has a high viscosity (Jauniaux et al., 1991c). Primitive erythrocytes are characterized by the presence of embryonic globins with high oxygen affinities (Manning et al., 2017). When the definitive erythropoiesis starts in the fetal liver around 8 weeks of gestation (Baron et al., 2013), the number of nucleated red cells drops (Jauniaux et al., 1991b) and there is a switch to fetal/adult globins. Equally, serial reconstructions of embryos at this stage of development reveal that the connection between the descending aorta and the umbilical circulation is extremely narrow (Corner, 1929). Originally interpreted by Corner as evidence that the two circulations differentiate independently *in situ*, the constriction must limit blood flow to the placenta, possibly protecting the forming heart from the high resistance of the placental circulation in the process. These limitations to the establishment of both of the placental circulations during the first 2 months of gestation support the concept that the developing embryo and its placenta are protected from excessive oxygen exposure during the sensitive period of organogenesis (Jauniaux et al., 2003b). The combination of anatomical and physiological barriers provides the embryo with what is strictly necessary to its development (Jauniaux et al., 2003c).

The chorionic circulation develops progressively during the 3rd month of pregnancy and coincides with the establishment of the arterial inflow into the intervillous space of the placenta (Jauniaux et al., 2000). How the onset of the two circulations is co-ordinated is not known, but cues may be altered pressure differentials across the villous membrane following expansion of the intervillous space, the associated increase in oxygenation or changes in local cytokine or hormone production by the trophoblast secondary to an increase in shear stress at the villous surface.

## Establishment of the maternal arterial circulation to the placenta

Contrary to many of the standard embryological textbook accounts, the maternal arterial circulation to the human placenta is not fully established in normal pregnancies until toward the end of the first trimester (Jauniaux et al., 2000). During the early weeks of pregnancy the maternal spiral arteries that will ultimately supply the placenta undergo extensive remodeling that involves the loss of elastin and smooth muscle from their walls and dilation of the terminal segments that open into the intervillous space (Harris, 2010). This remodeling ensures a continuous high volume of maternal blood flow through the placenta after onset of the circulation at the end of the first trimester, but at a low velocity and pressure that avoids mechanical damage to the villous tress and allows an adequate transit time for maternal-fetal exchange (Burton et al., 2009).

**Figure 3:**
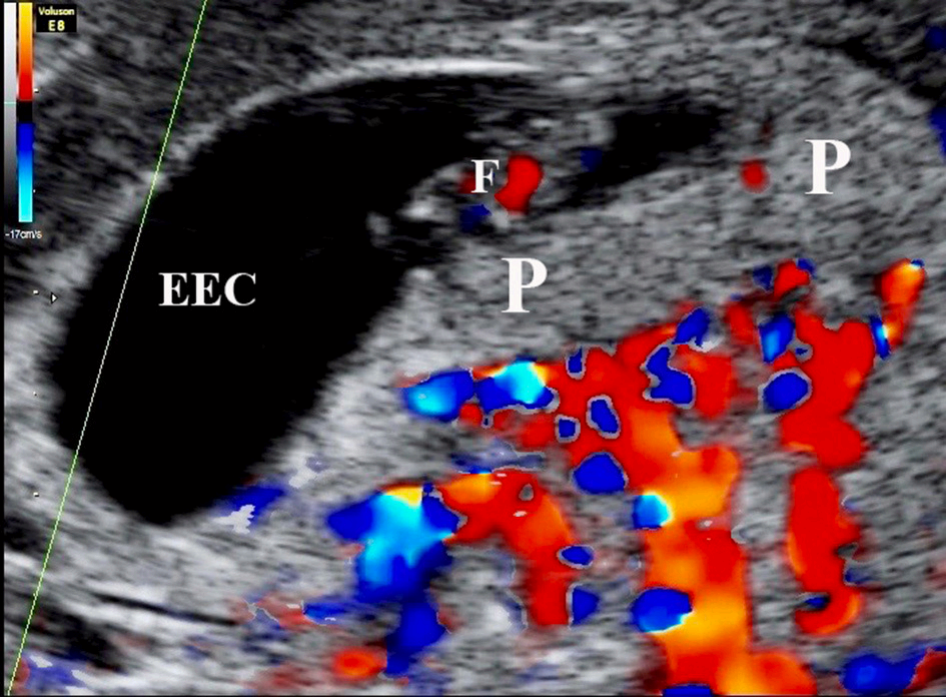
Color Doppler mapping of the utero-placental circulation at 7 weeks 2 days of gestation showing the absence of circulation within the placenta (P). ECC, exocoleomic cavity; F, Fetus.

Remodeling is achieved through the actions of the extravillous trophoblast (EVT) cells that migrate from the placenta into the underlying decidua, both through the stroma and down the lumens of the spiral arteries as interstitial and endovascular trophoblast, respectively. The magnitude of the endovascular migration is such that the mouths of the arteries are effectively plugged for most of the first trimester (Hustin and Schaaps, 1987; Burton et al., 1999), with only a network of intercellular spaces connecting the arterial lumen to intervillous space (Figure 3). Plasma may pass at a slow rate through these spaces, but erythrocytes are largely excluded. Consequently the placenta is filled with a clear fluid for most of the first trimester (Hustin et al., 1988), and the oxygen concentration is relatively low (Jauniaux et al., 1999, 2000), although there is no evidence that the placental tissues are hypoxic (Cindrova-Davies et al., 2015). Histotrophic nutrition from the endometrial glands contributes to support of the conceptus at this time (Burton et al., 2002), and provides a rich supply of glucose to maintain glycolysis (Burton et al., 2017).

Toward the end of the first trimester the network of intercellular spaces gradually coalesces and dilates to form channels through the endovascular “plugs,” allowing maternal arterial blood to flow freely into the intervillous space (Burton et al., 1999; Roberts et al., 2017). This development has been documented using ultrasonography, but is best confirmed by the three-fold rise in oxygen concentration within the placenta between weeks 10 and 12 of gestation measured using an intra-arterial probe (Jauniaux et al., 2000). This rise in oxygenation, and/or the increase in shear stress at the villous surface caused by the maternal blood flow, which at this stage is still pulsatile (Collins et al., 2012), may stimulate the release of local vasodilators, such as nitric oxide (NO) from the syncytiotrophoblast. Such agents might in turn act on the closely approximated fetal capillaries, hence opening the chorionic circulation. This is an attractive hypothesis but it remains to be tested; the syncytiotrophoblast contains nitric oxide synthase (NOS) (Myatt et al., 1993), but as yet there are no data available indicating the sensitivity of this enzyme to shear stress.

Deficiencies in spiral artery remodeling are associated with a spectrum of complications of pregnancy, including miscarriage, pre-eclampsia, fetal growth restriction (FGR), and pre-term labor (Brosens et al., 2011). As will be discussed later, it is possible that defects in cardiac myogenesis arise secondary to poor placentation through malperfusion of the placenta and the generation of excessive intra-placental oxidative stress.

## Assessment of the chorionic circulation *in vivo*

**Figure 4:**
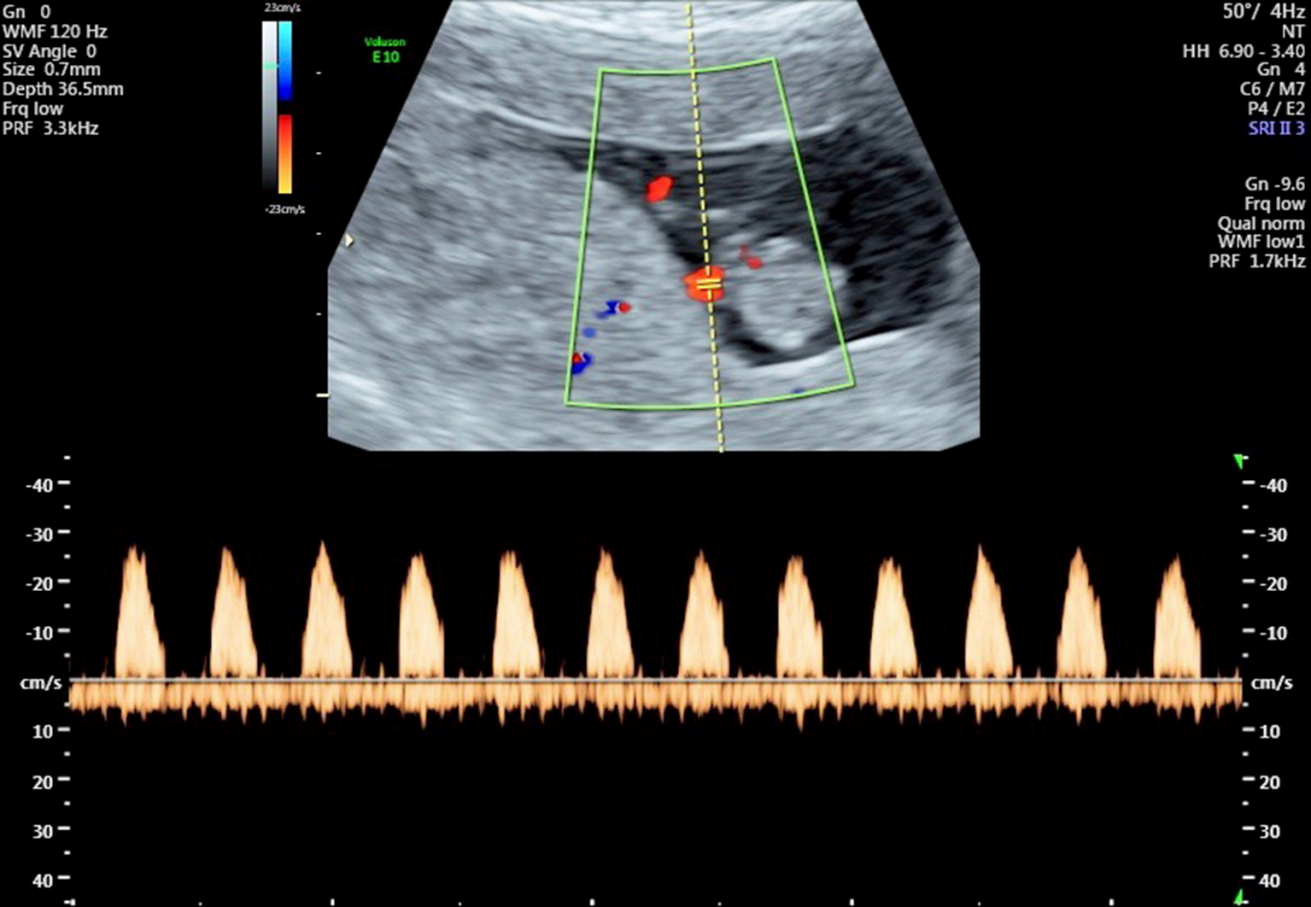
Flow velocity waveforms from the umbilical artery at 10 weeks and 6 days showing the absence of end-diastolic flow in all cardiac cycles.

**Figure 5:**
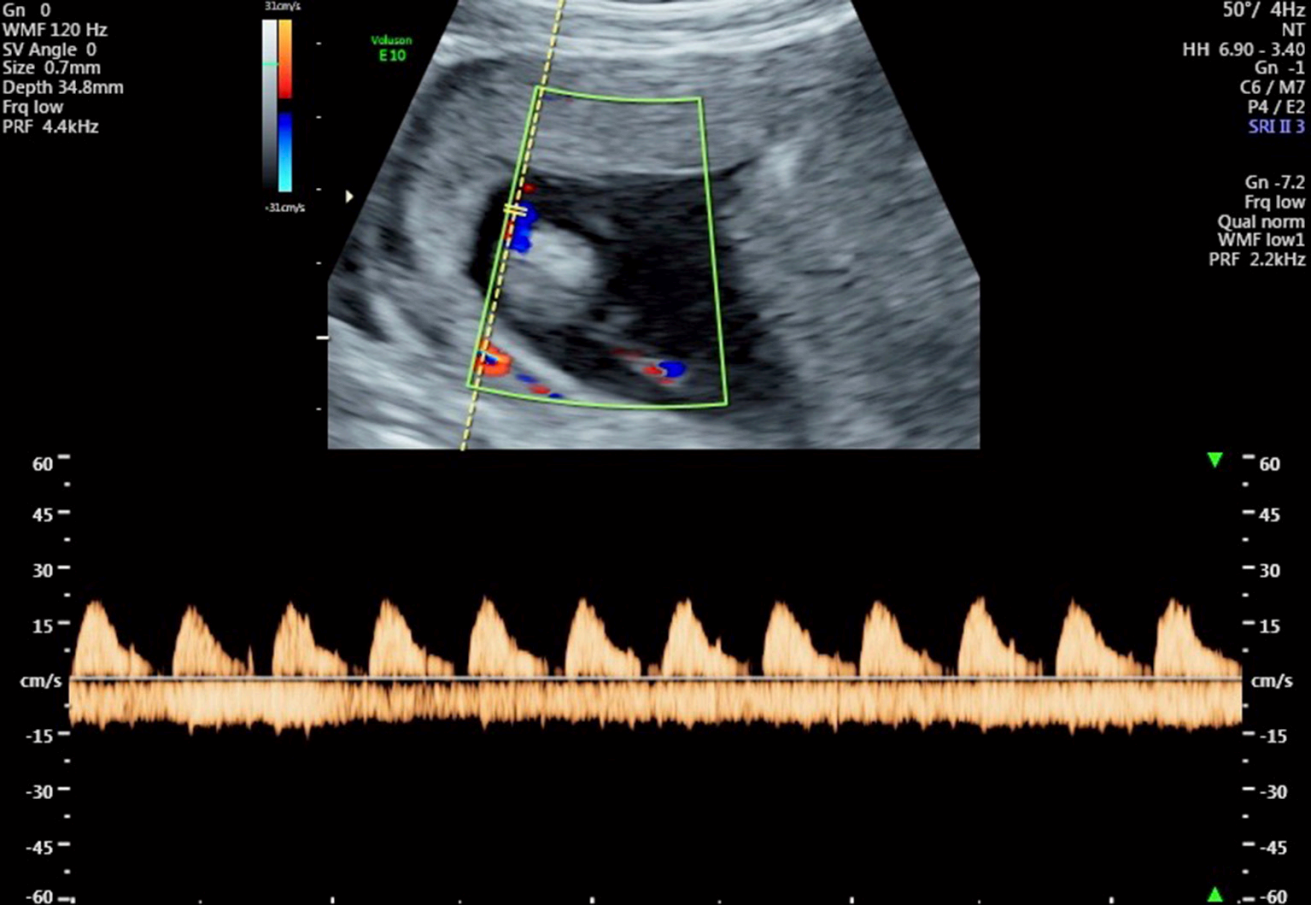
Flow velocity waveforms from the umbilical artery at 13 weeks and 1 day showing partial end-diastolic flow. In some cardiac cycles there is flow throughout almost the entire diastolic phase, indicating a reduction in vascular resistance in the umbilico-placental vascular bed.

The advent of Doppler ultrasound imaging has enabled the characteristics of the chorionic circulation to be followed from early in pregnancy by monitoring waveforms in the umbilical arteries and its main intra-placental branches (Jauniaux et al., 1991c, 1992). Early studies have shown a relationship with advancing gestation between the changes in flow velocity waveforms on Doppler imaging and the morphological development of the villous trees and their contained capillary networks (Loquet et al., 1988; Jauniaux et al., 1991b; Huisman et al., 1992; Mercé et al., 1996). These studies all showed major changes in the resistance to blood flow in the umbilico-placental circulation around the transition between the first and second trimesters. Until week 10 of gestation there is an absence of end-diastolic flow in the umbilical circulation, indicative of a high resistance (Figure 4). The end-diastolic flow gradually appears in the umbilical circulation between 12 and 14 weeks of gestation (Figure 5), indicating a decrease in resistance and allowing continuous perfusion of villi of the definitive placenta during the entire cardiac cycle (Jauniaux et al., 1991b). The mechanism by which a reduction in vascular impedance through the feto-placental circulation occurs during the second trimester is not known. Between 9 and 15 weeks, villous tissue NO and cyclic guanosine monophosphate (cGMP) concentrations are positively correlated with umbilical artery impedance, suggesting that these molecules modulate the reduction in resistance within the umbilico-placental circulation that occurs during the first trimester of pregnancy (Lees et al., 1998). The passage of fetal blood through an anatomically high-resistance circuit may possibly lead to stimulation of endothelial NOS activity, thus maintaining vasodilatation within the umbilico-placental circulation until further elaboration of the villous tree occurs. Alternatively, it may be due to the replacement of nucleated erythrocytes by their more easily deformable non-nucleated counterparts (Jauniaux et al., 1991b).

Peak systolic velocity increases more than three-fold progressively between 7 and 10 weeks, and the umbilical resistance index decreases dramatically at ~10 weeks (Mäkikallio et al., 2005). As the diameter of the outflow tract increases over the same period, the authors concluded that there must be a considerable increase in the umbilico-placental volume of blood flow at the end of the first trimester. This increase coincides temporally with completion of cardiac organogenesis (Gittenberger-de Groot et al., 2013), and with the start of diphasic diastolic filling of the atria, indicative of a more active umbilico-placental circulation and an increased venous return to the heart (Mäkikallio et al., 2005). Since the placental vasculature shows only gradual changes in vessel length and diameter across this period (Jauniaux et al., 1991a), these haemodynamic changes must presumably reflect opening of either the connection between the aorta and the umbilical arteries, or the existing capillaries within the villi and a reduction in the number of nucleated erythrocytes and thus overall blood viscosity.

During normal pregnancy the gradual reduction in the resistance to blood flow in the umbilical artery continues during the second and third trimesters, reflecting enlargement of the placental vasculature and/or the elaboration of peripheral villi and the creation of more parallel circuits. In pregnancies complicated by severe FGR, resistance may increase again during the second half of gestation, leading to absent or even reversed end-diastolic flow. These have pathological correlates, as follows.

## Placental pathology and fetal cardiac development

Epidemiological data have linked an individual's risk of cardiac disease with different attributes of their placenta, including its weight, surface area, and thickness (Barker et al., 2010, 2012; Eriksson et al., 2011). Whilst it might be expected that alteration of placental development impacts on fetal cardiac development, as it does with many other organs, there is limited experimental evidence of a direct linkage. Manipulation of the peroxisome proliferator-activator receptor (PPAR) or mitogen activated protein (MAP) kinase pathways in the placenta alone has been reported to cause cardiac defects in mice (Barak et al., 1999; Adams et al., 2000). The mechanisms remain unknown, and further research is required to fully assess the extent of what has been referred to as the heart-placenta axis (Linask, 2013).

Associations between cardiac defects and placental abnormalities have been reported following pathological examination of the placenta after delivery. Thus, hypoplastic left heart syndrome has been linked to a reduced placental weight, villous immaturity with reduced vascularization and vasculo-syncytial membranes, and increased fibrin deposition (Jones et al., 2015). Whether the effects are mediated through reduced transport efficiency, altered endocrine function—there was an increase in placental leptin levels, or haemodynamic changes in the umbilical circulation is not known. A trend toward a reduced placental volume has been also reported in a prospective trial of complex congenital heart disease using MRI, and the lack of statistical significance may be due to the relatively small sample size (Andescavage et al., 2015). A strong association with FGR has been recently found in women carrying a fetus with congenital heart disease of having a higher risk (odds ratio 3.32; 95% CI−2.39 to 4.56) of a birth weight lower than the 3rd centile (Ruiz et al., 2016). Eccentric insertion of the umbilical cord has also been associated with a higher risk of congenital heart disease, (odds ratio of 2.33–3.76) (Albalawi et al., 2017). The main defects were conotruncal and left and right heart disease, and again there was an association with FGR. Finally, fetal thrombotic vasculopathy has been associated with a six-fold increase in congenital heart disease (Saleemuddin et al., 2010).

Separating the effects of placental function from those of aberrant umbilical haemodynamics is difficult in the human situation. Experimental manipulations in animal models have shown that the haemodynamics within the vitelline or umbilical circulations can have a major impact on the differentiation of cardiomyocytes and heart development (Kowalski et al., 2014; Midgett et al., 2017). A common theme in many of the pathological studies is that of FGR. As described earlier, these pregnancies are often associated with absent or reversed end-diastolic umbilical arterial flow, indicative of raised placental vascular resistance (Arbeille, 1997; Soregaroli et al., 2002). Morphological studies searching for correlates following delivery have focused on the arteries within the stem villi, which are believed to be the principal resistance vessels in the placental circulation. While there is no reduction in the number of vessels within the villi (Jackson et al., 1995), suggesting that vasculogenesis and angiogenesis were initially normal, there have been consistent reports of medial hypertrophy and a reduction in luminal caliber (Fok et al., 1990; Salafia et al., 1997; Mitra et al., 2000).

We have recently demonstrated that these changes can be mimicked *in vitro* by exposing arterial explants taken from term placentas to oxidative stress (Lu et al., 2017). Induction of stress causes downregulation of the enzyme cystathionine-γ-lyase that generates the gasotransmitter hydrogen sulfide. Hydrogen sulfide maintains vascular smooth muscle cells in a differentiated state, and blocking its production causes the cells to adopt a pathological proliferative phenotype. As discussed earlier, pregnancies complicated by FGR are commonly associated with poor placentation and deficient remodeling of the maternal spiral arteries (Brosens et al., 2011). The aberrant remodeling is a powerful inducer of placental oxidative stress, either through increased shear stress at the villous surface or through malperfusion and ischaemia-reperfusion type injury (Burton and Jauniaux, 2011). We therefore speculate that the change in placental vascular resistance and umbilical arterial waveforms is secondary to increasing placental oxidative stress in later pregnancy, although the pathophysiological seeds are sown during the first trimester. The effect on the fetal heart is currently unknown, but it might be speculated that the increased resistance in the umbilico-placental circulation causes a degree of hyperplasia.

## Conclusions

Development of the heart and the placenta are likely to be closely interlinked through several mechanisms. Firstly, failure of correct placentation results in fetal growth restriction, and an impaired nutrient supply or adverse fetal endocrine environment may have non-specific effects on the growth and differentiation of many organ systems (Burton et al., 2016). A recent study in a non-human primate has indicated that growth restriction mimics accelerated aging of the heart (Kuo et al., 2017), but a detailed consideration of these effects is beyond the scope of the current review. Secondly, cardiac and placental abnormalities may co-exist through polymorphisms in genetic developmental pathways common to both organs, in particular those regulated by Wnt/ß-catenin signaling, or through a lack of key micronutrients, such as folate (Linask, 2013). During evolution, gene networks underpinning development of other organs were recruited by the placenta (Cross et al., 2003), and so basic processes, such as cell adhesion and angiogenesis, are shared with the heart. Hence, disruption of integrin alpha 4 or of its ligand vascular cell adhesion molecule (VCAM-1) is associated with both major placental and cardiac abnormalities. In the placenta there is a failure of fusion of the allantois with the chorion, whereas in the heart there is a aberrant development of the epicardium and the coronary vessels (Kwee et al., 1995; Yang et al., 1995). A similar situation arises with Hand-1 (McFadden et al., 2005), with the embryos of murine knockouts dying at day E8.5 through placental failure. Thirdly, major abnormalities, such as transpositions, may arise if the placenta fails to function as a selective barrier to xenobiotics and other teratogens. Lastly, and the focus here, is that aberrant haemodynamics in the umbilico-placental circulation may influence cardiac development. As this circulation is only established toward the end of the period of cardiac organogenesis, it is more likely that placental problems influence cardiac differentiation biomechanically later in pregnancy when the placenta receives ~40% of fetal cardiac output. Changes in the umbilical vascular resistance through thrombotic vasculopathy or medial hyperplasia in the stem villus arteries could affect differentiation of the cardiomyocytes, resulting in hypo- or hyperplastic syndromes. More research is required to test this hypothesis, but if proved correct then interventions, such as hydrogen sulfide donors, may prove to be beneficial (Lu et al., 2017).

## Author contributions

All authors listed have made a substantial, direct and intellectual contribution to the work, and approved it for publication.

### Conflict of interest statement

The authors declare that the research was conducted in the absence of any commercial or financial relationships that could be construed as a potential conflict of interest.
